# New Trends in Paracoccidioidomycosis Epidemiology

**DOI:** 10.3390/jof3010001

**Published:** 2017-01-03

**Authors:** Roberto Martinez

**Affiliations:** Division of Infectious Diseases, Department of Internal Medicine, Ribeirão Preto Medical School, Universidade de São Paulo, Sao Paulo 14049-900, Brazil; rmartine@fmrp.usp.br; Tel.: +55-16-3602-2468

**Keywords:** paracoccidioidomycosis, *Paracoccidioides brasiliensis*, *Paracoccidioides lutzii*, epidemiology

## Abstract

Paracoccidioidomycosis is a systemic fungal disease occurring in Latin America and more prevalent in South America. The disease is caused by the dimorphic fungus *Paracoccidioides* spp. whose major hosts are humans and armadillos. The fungus grows in soil and its infection is associated with exposure to the rural environment and to agricultural activities, with a higher risk in coffee and tobacco plantations. Population studies assessing the reactivity to *Paracoccidioides* spp. antigens by intradermal reaction or serological tests have detected previous subclinical infections in a significant proportion of healthy individuals living in various endemic countries. Paracoccidioidomycosis-disease is manifested by a small minority of infected individuals. The risk of developing the disease and its type of clinical form are related to the personal and life style characteristics of infected individuals, including genetic background, age, sex, ethnicity, smoking habit, alcohol drinking, and eventual cellular immunosuppression. Brazil, Colombia, Venezuela, Argentina, and Ecuador have endemic areas that had already been defined in the 20th century. The incidence of paracoccidioidomycosis can be altered by climate phenomena and mainly by human migration and occupation of poorly explored territories. In Brazil, the endemy tends to expand towards the North and Center-West around the Amazon Region.

## 1. Introduction

Paracoccidioidomycosis is an endemic fungal infection occurring on the American continent from 23° N to 23° S, although with higher prevalence in South America. The infection was first described in 1908 by Adolpho Lutz, who also isolated the microorganism causing the disease from material obtained from oral patient lesions [1]. For a long period of time the thermodimorphic fungus *Paracoccidioides brasiliensis*, an *Ascomycota* of the family *Ajellomycetaceae*, order *Onygenales*, was recognized as the only agent inducing the mycosis*.* Genotypic studies revealed variations of *P. brasiliensis*, which was assigned to the cryptic species PS1 (more common and isolated from different regions of South America), PS2 (isolates from Brazil and Venezuela), PS3 (isolates from Colombia), and PS4 (apparently limited to Venezuela) [2]. *P. lutzii*, a new species of the genus *Paracoccidioides*, was also identified by means of multilocus sequencing studies [2]. The geographic distribution of *P. lutzii* is more centered on the Center-West of Brazil, a region inhabited by many patients from whom the microorganism was isolated [3]. Data about pathogenicity or clinical manifestations are relatively unknown when compared to *P. brasiliensis*. Practically all information about the eco-epidemiology, infection, and disease concerning to *Paracoccidioides* spp. has been obtained in studies that did not distinguish between the species involved.

*Paracoccidioides* spp. grows saprophytically in the soil of the American continent but has been seldom isolated from nature, thus preventing the identification of the exact location and of the microniche characteristics of this fungus [4]. *P. brasiliensis* and *P. lutzii* have been recently detected by molecular methods in different soil samples from three distinct locations in Brazil [3]. The development of *Paracoccidioides* spp. in the natural environment, supposedly in its mycelial form, has been associated with humid regions, medium to high pluviosity, mild temperatures, and the presence of rivers and forests [5]. More recent studies have reported that *Paracoccidioides* spp. infection and disease also occur in areas of agricultural crops [6,7] and that armadillos infected with the fungus live in territories with sandy and acidic soil, disturbed natural vegetation, and the presence of rivers [8].

The human species and nine-banded armadillos are the main accidental hosts of *Paracoccidioides* spp. which can be infected in the rural or periurban environment [8,9]. In the rural environment, conidia apparently enter through the respiratory route and reach the lungs, where the infection is usually controlled by the immune cellular response, but may leave behind cicatricial focal points with latent yeast cells. The infection is asymptomatic or manifests clinically with nonspecific symptoms and only a very small percentage of infected individuals progress to the disease [10]. There are no reports of epidemic outbreaks of paracoccidioidomycosis or transmission of *Paracoccidioides* spp. between individuals.

Paracoccidioidomycosis-disease manifests in two major clinical forms which are distinguished on the basis of demographic and clinical aspects and of the immunological response of the patients [11]. The acute/subacute (“juvenile”) form predominantly occurs in children and young adults who have a Th2 type cell immune response which is inadequate to control the fungal infection. The disease develops a few weeks or months after exposure to *Paracoccidioides* spp., permitting the identification of areas of acquisition of this fungal infection. The most common clinical manifestation in this form of the disease is generalized or intra-abdominal lymphadenomegaly frequently accompanied by lesions of the skin, the oral and intestinal mucosa and the bone, and hepatosplenomegaly. In contrast, the chronic (“adult”) form of paracoccidioidomycosis which affects about 80% to 95% of cases, usually occurs after 30 years of age and is limited to lesions in the lungs and upper airways and commonly on the oral mucosa and on the skin adjacent to the mouth and nose. Some patients have lesions due to dissemination of the fungus to the adrenals and brain. The period of incubation in the chronic form of the mycosis is uncertain, but the disease is considered to be likely to occur many years after exposure to *Paracoccidioides* spp.

The present review deals with the epidemiological aspects of paracoccidioidomycosis-disease related both to its acquisition and outcome. The results of studies about the infection of human and animal populations that do not show illness are also presented in order to show the extension and degree of endemicity of the geographic areas occupied by *Paracoccidioides* spp. The bases of the epidemiology of paracoccidioidomycosis were already known by the mid-twentieth century [1]. However, epidemiological modifications have been observed over the last three decades and have been mainly related to changes of interaction between the human species and the natural environment.

## 2. Prevalence, Incidence, Lethality, and Mortality

In view of the lack of continued recordings of paracoccidioidomycosis cases, the prevalence of this mycosis has been estimated on the basis of reported case series and, in Brazil, also based on hospitalization and mortality data [11,12,13,14,15,16,17,18,19,20,21,22,23,24,25,26,27,28,29,30,31,32,33,34,35,36,37] (Table 1). Several thousand cases are presumed to be diagnosed each year in the vast territory where this fungal disease is endemic. About 80% of the patients acquire the disease in Brazil and most of the remaining ones acquire it in other South American countries, mainly Colombia, Venezuela, Argentina, and Ecuador. A total of 168 deaths per year due to paracoccidioidomycosis were recorded in Brazil from 1996 to 2006 [36]. By assuming that the lethality rate of this fungal disease is 3% to 5%, the number of cases of paracoccidioidomycosis in Brazil is estimated to range from 3360 to 5600 per year.

Previous studies have estimated the annual incidence of paracoccidioidomycosis-disease at 0.96 (São Paulo, Brazil), 0.71 (Rio de Janeiro, Brazil), and 0.90 (Santa Maria, Southern Brazil) per 100,000 inhabitants [21,38]. Between 1980 and 1999, the incidence in the Ribeirão Preto region, Southeastern Brazil, ranged from 1.5 to 3.7 cases/100,000 inhabitants/year [6]. In Colombia, the cases detected between 1970 and 1999 permitted researchers to estimate the incidence of the mycosis between 0.5 and 2.2/100,000 inhabitants/year, with the six municipalities with the highest incidence having rates of 0.8 to 3.1/100,000 inhabitants/year [29]. These data led to an estimated occurrence of one to four cases/100,000 inhabitants per year in geographic areas with stabilized endemicity. In contrast, in a hyperendemic area more recently installed in the state of Rondônia, West Amazon Region, the mean incidence reached 9.4/100,000 inhabitants/year and some municipalities in the Southeastern part of this state had an incidence close to 40 cases/100,000 inhabitants/year [14].

The deaths due to paracoccidioidomycosis are caused by extremely disseminated lesions, respiratory insufficiency, adrenal insufficiency, and several other complications, and may occur a long time after antifungal treatment. In two case series from different endemic Brazilian areas, lethality reached 6.1% and 7.6%, respectively [14,39]. However, when assessed at the end of treatment in patients who did not require hospitalization, lethality was zero [40]. A total of 1853 deaths due to paracoccidioidomycosis were recorded in Brazil between 1986 and 2006, representing 51% of the total number of deaths due to fungal infections [36]. During the period from 1980 to 1995, the mean annual mortality rate per million inhabitants ranged from 0.20 (Northeastern region) to 2.59 (Southern Brazil), and from 2002 to 2004 it ranged from 0.20 (Northeast) to 2.10 (Center-West) [36,41]. The mortality rate is decreasing in the Southern and Southeastern regions of Brazil, while it is increasing in the Northern region, where the states of Rondônia and Acre had rates of 8.2 and 5.6/1,000,000 inhabitants, respectively, in 2002–2004 [36]. Analysis of the data for the five major regions of Brazil reveals that the Brazilian Center-West and North regions simultaneously have the highest rates of hospitalization and mortality (Table 1), suggesting that the western side of Brazil is currently an important endemic area of paracoccidioidomycosis.

## 3. Predisposing and Modulating Factors

The progression from infection to disease due to *Paracoccidioides* spp. depends on the type of immunological response of the host, but is also influenced or modulated by his genetic background, demographic characteristics, and life conditions and style.

### 3.1. Gender

At prepubertal age, paracoccidioidomycosis equally affects both genders, but after puberty about 75% to 95% of the patients are men [17]. Although women are infected with *Paracoccidioides* spp. as much as men, they are less likely to develop paracoccidioidomycosis apparently because their circulating estrogens inhibit the transformation of the aspirated conidia into yeast cells and also modulate the cell immune response against the fungus [42]. The greater proportion of men involved in agricultural activities in endemic areas is an additional factor explaining the predominance of males in case series of this fungal disease. Paradoxically, women of fertile age who progress to paracoccidioidomycosis-disease tend to have more disseminated fungal lesions and clinical signs and symptoms corresponding to the acute/subacute form when compared to men [11]. This fact may be attributed in part to the hormonal and immunity changes that occur during pregnancy, which has been associated with the onset or reactivation of paracoccidioidomycosis [43].

### 3.2. Age

The disease caused by *Paracoccidioides* spp. has been observed over a wide age range, from two-year-old children to the elderly, although most patients are 30 to 60 years old [15,20]. For reasons that are still unknown, but that may possibly be related to immunological-hormonal interactions, the clinical manifestations of paracoccidioidomycosis are strongly associated with the age range of the patient. Children of both sexes exclusively have the acute/subacute form of the mycosis, which also predominates among young adults up to 30 years of age. Starting from the third decade of life, the chronic form of the mycosis is more prevalent [6].

### 3.3. Ethnicity

Racial predisposition to the development of paracoccidioidomycosis has not been detected. However, an analysis of the mycosis in a miscegenated population of Southern Brazil has revealed that blacks and mulattoes more frequently show more disseminated lesions of the acute/subacute form than white patients [11]. The association of the clinical signs and symptoms of the mycosis with ethnicity may be due to the variation of genetic constitution and possibly to differences in living conditions.

### 3.4. Genetic Variability

Comparison of the histocompatibility system of patients with paracoccidioidomycosis to that of healthy subjects has shown that the former have a higher proportion of HLA-A9 and HLA-B13 antigens and the C4B*-Q0 antigen of the class III major histocompatibility complex was associated with the chronic form of the mycosis [44]. Evaluation of single nucleotide polymorphisms of the genes coding for cytokines has revealed a higher frequency of the IL12RB1 641AA genotype in men with the chronic multifocal form of this fungal disease [45]. These studies suggest that the variation of the genetic background of individuals and of populations living in different endemic areas may be related both to progression of the infection to paracoccidioidomycosis-disease and to its clinical expression.

### 3.5. Exposure in Rural Areas

In case series from different endemic countries, the great majority of patients reported current or past contact with the rural environment due to their profession, residence, or both [25,31]. Exposure at the periphery of cities where the urban and rural zones overlap has also been reported by the patients. Paracoccidioidomycosis is an occupational disease of farmers and of other professionals who are exposed to aerosol containing soil particles. Working with coffee and tobacco crops has been associated with an increased risk to acquire infection and disease due to *Paracoccidioides* spp. [6,46].

### 3.6. Smoking, Alcohol Drinking, and Life Style

Smoking and the pulmonary changes caused by this habit are strongly associated with later lung involvement by *Paracoccidioides* spp. In the chronic form of the mycosis, 90% of the patients are smokers and their risk to develop this disease is 14 times higher among smokers than nonsmokers [23,47]. The intake of distilled alcoholic drinks with mean alcohol quantities exceeding 50 g per day also favors the onset of paracoccidioidomycosis [47]. It is presumed that a low socioeconomic level, living at the periphery of the urban region, and malnutrition may also facilitate the development of the mycosis.

## 4. Relationship with Cancer, AIDS, and Chronic Infections

The factors predisposing to paracoccidioidomycosis apparently favor the onset of other diseases in the same patient. Neoplasias, tuberculosis, Chagas disease, leishmaniasis, leprosy, and strongyloidiasis may be more frequent in cases of paracoccidioidomycosis, occurring before, after, or simultaneously with this mycosis [6]. The disease is more commonly associated with tuberculosis which affects up to 15% to 20% of the patients [48]. Neoplasias, usually of the carcinoma type, is involved in 0.16% to 14.1% of paracoccidioidomycosis cases, arising at varying times, particularly after antifungal treatment of patients with the chronic form of the disease [23,49]. 

Opportunistic disease due to *Paracoccidioides* spp. was observed in a few dozen cases in which paracoccidioidomycosis arose concomitantly with or after the diagnosis of hematological or solid organ neoplasias [23,49]. This mycosis has been observed in immunosuppressed patients after renal transplantation [50], or in patients with immunological failure due to genetic changes [51]. In endemic areas of Brazil, about 1.5% of AIDS patients have opportunistic paracoccidioidomycosis, usually with disseminated lesions [52,53], and this mycosis was related to 1.4% of deaths in AIDS cases [36]. HIV coinfection has been detected in about 5% of patients with paracoccidioidomycosis [52], but its prevalence is currently decreasing due to the ample and early use of antiretroviral therapy.

## 5. Paracoccidioidomycosis-Infection

Attempts to isolate *Paracoccidioides* spp. from nature are usually fruitless, a fact that has led to other methods for the assessment of the territorial extension occupied by this microorganism and of the factors related to the infection that precedes paracoccidioidomycosis. This infection is commonly asymptomatic, non-progressive, and has been recognized on the basis of a few case reports and of the investigation of delayed hypersensitivity and of anti-*Paracoccidioides* spp. antibodies in healthy human populations and in animals.

The clinical manifestations, rarely observed during the early weeks after exposure to *Paracoccidioides* spp., correspond to the formation of the primary fungal complex and consist of fever, respiratory symptoms, and chest X-rays revealing focal pulmonary lesions and lymphadenomegaly in the pulmonary hilum. These manifestations, particularly the radiographic alterations, can be different from those observed in paracoccidioidomycosis-disease and are self-limited, a fact that hampers the recognition of the *Paracoccidioides* spp. infection [10]. A few cases progress to the acute/subacute form of paracoccidioidomycosis-disease [54]. Further evidence of asymptomatic or oligosymptomatic infection with *Paracoccidioides* spp. is observed in some patients with fibrotic pulmonary nodules identified as paracoccidioidomas [55].

The detection of paracoccidioidomycosis infection in healthy individuals is usually based on intradermal application of paracoccidioidin, glycoprotein gp43, and other fungal antigens, and on the measurement of skin reactivity after 24 to 48 h. The results of numerous studies have been reported in two reviews [56,57]. The rates of infection are not directly comparable because of the methodological differences between studies, mainly concerning the use of different antigens. Another limitation is the possibility of cross-reaction with *Histoplasma capsulatum* antigens [58]. Surveys using intradermal reaction to *Paracoccidioides* spp. antigens have shown similar rates of infection with this fungus among men and women [57,59]. Children in the first decade of life may be positive to the test, but the rate of infection usually increases with age, reaching maximum values after 30 years of age [59,60]. Infection with *Paracoccidioides* spp. has been associated with residence and professional occupation in the rural area and may be favored by contact with coffee cultures, armadillos, and bats [7,46,61].

**Figure 1:**
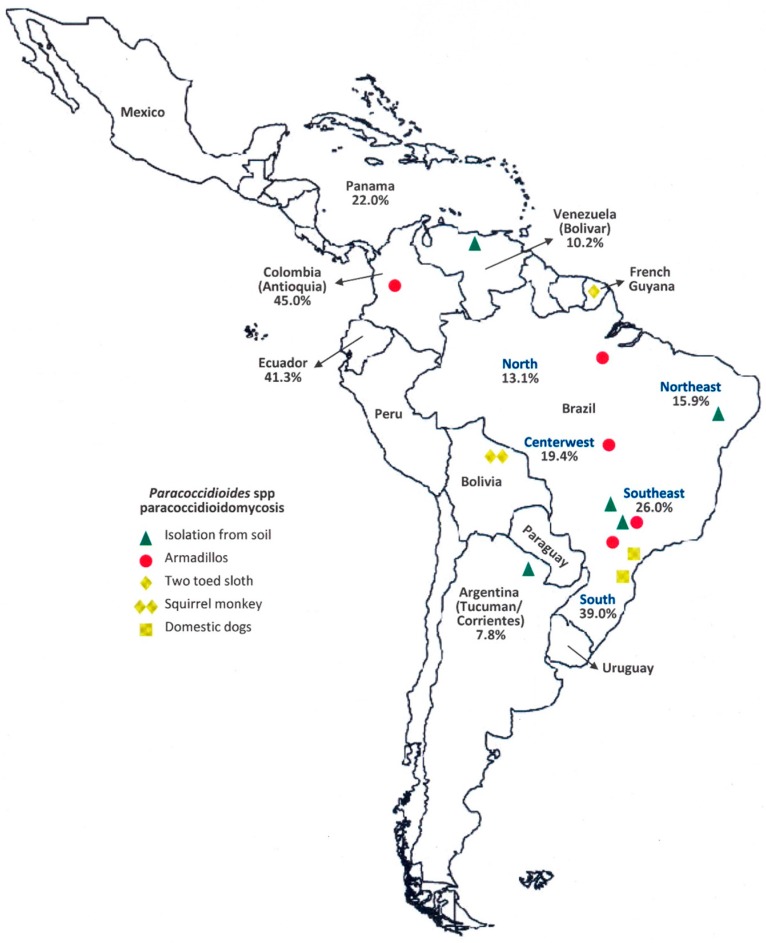
Rate of the *Paracoccidioides* spp. infection determined by intradermal test in general populations according to geographic area [7,31,38,56,57,59,60,61,62,63]. Comparatively, the marks show the places where this fungus was isolated form soil [4] or where captured animals has paracoccidioidomycosis: armadillos [8,9], two-toed sloth [73], squirrel monkey [72], and domestic dogs [70,71].

Figure 1 shows the median rates of paracoccidioidomycosis-infection exclusively obtained in studies of general populations in various Latin-American countries [7,31,38,56,57,59,60,61,62,63]. Individuals reacting to *Paracoccidioides* spp. were detected in all regions investigated, suggesting that significant parcels of the populations between Northern Argentina and Panama had been infected with this microorganism. More recent studies have detected rates of infection ranging from 4% to 47% in Brazil, Argentina, and Venezuela [59,60,62], values comparable to those of surveys held during the last century.

In Brazil, anti-*Paracoccidioides* spp. antibodies have been detected in 27% of blood donors in the state of Paraná and in 5% of Indians in the state of Minas Gerais [64,65]. These studies suggest previous asymptomatic infection with this fungus, but may also be limited by possible cross-reactivity with other agents of fungal infection.

Intradermal and serological tests conducted on domestic and free or captive wild animals have revealed that various mammals and birds may be infected with *Paracoccidioides* spp. [66,67,68]. It was observed that the rate of infection is higher in dogs from the rural area than in dogs from the urban zone and higher in rabbits raised free than in caged animals [66,69]. Part of the animals reacting to the tests was sacrificed and *Paracoccidioides* spp. was not detected in their viscera, suggesting effective control of the fungal infection [69]. On the other hand, there are reports of paracoccidioidomycosis-disease among domestic dogs living in the Brazilian Southeast and South [70,71]. With the exception of armadillos [8,9], this fungal disease was rarely observed in the other wild animals: a squirrel monkey [72], and a two-toed sloth [73] (Figure 1).

## 6. Consolidated Endemic Areas and Expansion of the Endemy

Autochthonous cases of paracoccidioidomycosis occur only in Latin America from Southern Mexico to Northern Argentina. Coccidioidomycosis and histoplasmosis are more prevalent in Mexico and Central America, where the number of cases of disease due to *Paracoccidioides* spp. is relatively small. In South America, the endemic areas of histoplasmosis and paracoccidioidomycosis are approximately superimposable, but the disease caused by *Paracoccidioides* spp. is more prevalent [74].

About 60 cases of paracoccidioidomycosis have occurred in European countries, the United States, Canada, Japan, Africa, and the Middle East [75,76,77]. However, the patients had visited or previously resided in South American countries and had presented paracoccidioidomycosis immediately or years after returning to their own country. This leads us to assume that *Paracoccidioides* spp. infection had occurred in South America.

**Figure 2:**
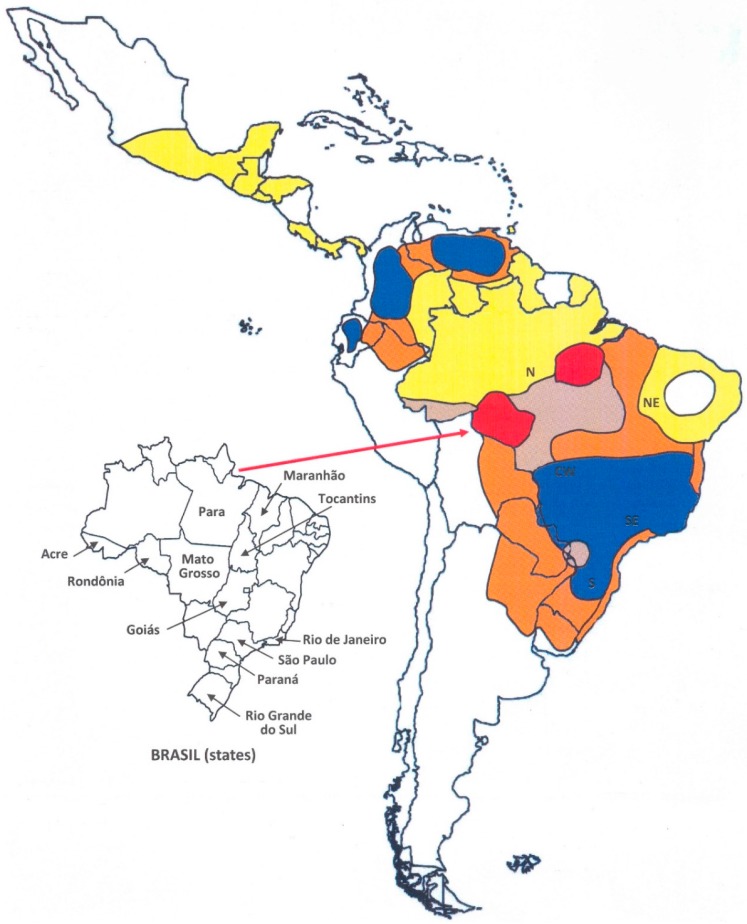
Geographic areas of paracoccidioidomycosis endemicity in Latin America: (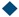) First recognized areas of high endemicity; (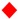) high endemicity observed since the last decades of the 20th century; (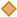) area with some recent evidence of increasing endemicity; (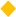) areas of moderate endemicity; (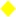) low endemicity; (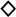) no areas or rare cases of paracoccidioidomycosis reported in these countries or regions.

Figure 2 shows the Latin American territories according to the degree of endemicity, as well as the area of expansion of paracoccidioidomycosis. Regions of high endemicity have been recognized since the 1950s in Brazil (Southeast, South, Center-West) [1,18], Colombia (Andes region) [5], and Venezuela (State of Bolivar and North region) [30]. Argentina (North) [28,38], Ecuador (Cuenca River valley) [31], and Paraguay (Oriental side) [32] have areas of moderate to high endemicity. Bolivia, Peru, and Uruguay have autochthonous cases, but the few data available do not permit a precise assessment of the endemic situation of paracoccidioidomycosis [33,35,38]. Southern Mexico, from the Gulf of Mexico to the Pacific Coast, is a territory of low endemicity, as is also the case for Central American countries [34].

Cases of acquired paracoccidioidomycosis currently exist in all major regions of Brazil, except the interior of the Northeast, where the climate is semi-arid. Because of its wide territorial extension and variety of biomes, together with human migration in the search for unexploited areas and agricultural frontiers, Brazil has experienced changes in the epidemiology of paracoccidioidomycosis over time (Figure 2). With a large number of new cases occurring each year, Southeastern Brazil was the first endemic area to be recognized. This was probably due to the extensive use of the rural environment for agriculture and animal husbandry, which exposed many workers to the risk of infection with *Paracoccidioides* spp. During the first half of the 20th century, coffee plantations occupied large parts of the Southeast, requiring the recruitment of workers from other countries. Many immigrants acquired paracoccidioidomycosis when working at coffee plantations and residing in the rural zone of the state of São Paulo. In a series of 1506 cases completed in 1955, 40% of the patients were immigrants, most of them from Japan and from European countries [18].

As a consequence of a governmental policy that promoted the occupation and colonization of frontier lands and of the Amazon Region and favored by the construction of highways, a movement of internal migration towards the Center-West and North occurred in Brazil during the second half of the 20th century for the establishment of agriculture and animal husbandry. This was probably the reason for the increased endemicity of paracoccidioidomycosis along the border of the Eastern Amazon Region (states of Tocantins, Pará and Maranhão) [15]. The same phenomenon of population migration and agricultural expansion occurred in the westernmost part of the Brazilian Amazon Region, leading to the onset of a hyperendemic area of paracoccidioidomycosis centered in the state of Rondônia and recognized since the 1990s [14]. In this region, the prevalence of malaria was similarly increased by the immigration [78]. Data regarding mortality and hospitalization due to paracoccidioidomycosis suggest that the southern portion of the Brazilian Amazon Region, extending to the frontier with Bolivia, tends to experience increased endemicity of the mycosis [36,37]. This region is currently undergoing a transformation similar to that of the regions mentioned earlier, involving increased population density, deforestation, and soil movement (Figure 2). Thus, the endemic area is expanding from the South and Southeast regions to the Center-West and North of Brazil.

## 7. Additional Causes of Epidemiological Modification

In addition to human migration and to the expansion of agricultural frontiers, there is evidence that climate and environmental changes and modifications in agricultural and social practices also have an impact on the occurrence of infection and disease induced by *Paracoccidioides* spp. An increased incidence of cases of acute/subacute paracoccidioidomycosis has been detected within one to two years after the observance of climate changes causing increased soil and air humidity [16]. The construction of major works involves soil digging, a flow of people, and a permanent environmental impact. An increase in *Paracoccidioides* spp. infection in the population of Northeastern Argentina was associated with the construction of the Yacyreta hydroelectric plant [63]. The construction of the Jirau and Santo Antônio hydroelectric plants in the Rio Madeira river attracted tens of thousands of people to the state of Rondônia and may have contributed to the increased number of cases of infection by this pathogen in the Brazilian western Amazon Region. Foz do Iguaçu, in Southern Brazil, showed a high prevalence of paracoccidioidomycosis in the past decade, perhaps related to the changes brought by the neighboring Itaipu hydroelectric plant and its respective artificial lake [22]. This suggests that the endemicity of paracoccidioidomycosis is increasing in this region, which may possibly include adjacent areas of Paraguay and Argentina (Figure 2) as well.

Changes in agricultural practices in stabilized areas of the endemy have generated the expectation of a long-term reduction of the incidence of paracoccidioidomycosis. The agricultural crops are being progressively mechanized, reducing worker exposure to *Paracoccidioides* spp. both because of the reduced number of persons involved and due to the lower risk compared to manual work. The extensive coffee plantations in the Brazilian Southeast have been replaced with sugar cane over the last three decades. The manual collection and drying of coffee grains implies a higher risk of aspiration of *Paracoccidioides* spp. conidia than the cutting of sugar cane, whose plantations undergo previous burning of the leaves which generates enough heat to destroy the microorganisms of the superficial layer of the soil [79]. Since most cases of paracoccidioidomycosis involve a long period of incubation, there is still no evidence of the reduction of the prevalence of the mycosis in areas where agricultural practices are changing.

In addition to the migration of a large portion of the rural population to the cities, child labor has been prohibited in Brazil since the 1990s. The reduced exposure of young people to *Paracoccidioides* spp. may be related to the progressive reduction of the incidence of acute/subacute paracoccidioidomycosis in case series of the Brazilian Center-West [27]. However, recent cases have been observed in children and young people who supposedly acquired the mycosis in the periphery of cities [80].

Apparently *Paracoccidioides* spp. grows in many territories and biomes of Latin America, and South America in particular. The fungal infection is closely related to exposure to the rural environment and to soil and vegetable crop manipulation. The existence of unexplored territories, such as those in the Amazon region shared by various South American countries, and human migration patterns following efforts to occupy uninhabited land with agriculture and animal husbandry leads to the prediction of a continued expansion of the endemic area of paracoccidioidomycosis, as already observed in the Brazilian North and Center-West. On the other hand, social and agricultural changes may lead to a reduced incidence of the mycosis in the future. The development of the disease and the type of clinical form manifested in infected people depends on the genetic background, demography, living conditions, and eventual immunosuppression of the host. New endemic areas usually have deficiencies in the structure of medical care, causing a greater social impact of paracoccidioidomycosis, which is a chronic disease requiring protracted treatment that can cause sequelae, disability, and death. 

## Figures and Tables

**Table 1:** Paracoccidioidomycosis endemy dimension in Brazil and other Latin America countries: reported cases number, hospitalization, and mortality rate.

Country/Geographic Area	Mortality Rate [36] ^a^	Hospitalization Rate [37] ^b^	Reported Cases (No.)	Annual Mean Number of Cases ^c^	Case Series References
Brazil	1.0	4.3	12,508		
North *	1.8	6.1	2375	159.9	[12,13,14]
*Northeast*	0.2	1.6	278	21.6	[15]
*Southeast*	1.0	5.7	6784	207.8	[11,16,17,18,19,20]
*South*	1.5	2.4	2169	140.6	[21,22,23,24]
*Midwest*	2.1	8.3	902	49.2	[25,26,27]
Argentina			110	110.0	[28]
Colombia			940	32.4	[29]
Venezuela			674	25.9	[30]
Ecuador			333	15.1	[31]
Paraguay			50	5.0	[32]
Peru			111	3.3	[33]
Mexico			93	3.1	[34]
Uruguay			48	1.1	[35]

^a^ Mortality rate/1,000,000 inhabitants/year (2002–2004); ^b^ Hospitalization rate/100,000 inhabitants/year (1998–2006); ^c^ Sum of the mean number of cases per year in the major series of cases in the same geographical area (1930–2012). * Fonts in italic indicate Brazilian geographical regions.
